# Long-term follow-up of MRI changes in thigh muscles of patients with Facioscapulohumeral dystrophy: A quantitative study

**DOI:** 10.1371/journal.pone.0183825

**Published:** 2017-08-25

**Authors:** Farzad Fatehi, Emmanuelle Salort-Campana, Arnaud Le Troter, Emilie Lareau-Trudel, Mark Bydder, Alexandre Fouré, Maxime Guye, David Bendahan, Shahram Attarian

**Affiliations:** 1 Centre de référence des maladies neuromusculaires et de la SLA, Centre hospitalier universitaire la Timone, Université Aix-Marseille, Marseille, France; 2 Aix-Marseille Université, Centre de Résonance Magnétique Biologique et Médicale, UMR CNRS 7339, Marseille, France; 3 Aix Marseille University, INSERM, GMGF, Marseille, France; 4 FILNEMUS, Marseille, France; University of Minnesota, UNITED STATES

## Abstract

Facioscapulohumeral muscular dystrophy (FSHD) is one of the most common hereditary muscular disorders. Currently FSHD has no known effective treatment and detailed data on the natural history are lacking. Determination of the efficacy of a given therapeutic approach might be difficult in FSHD given the slow and highly variable disease progression. Magnetic resonance imaging (MRI) has been widely used to qualitatively and quantitatively evaluate *in vivo* the muscle alterations in various neuromuscular disorders. The main aim of the present study was to investigate longitudinally the time-dependent changes occurring in thigh muscles of FSHD patients using quantitative MRI and to assess the potential relationships with the clinical findings. Thirty-five FSHD1 patients (17 females) were enrolled. Clinical assessment tools including manual muscle testing using medical research council score (MRC), and motor function measure (MFM) were recorded each year for a period ranging from 1 to 2 years. For the MRI measurements, we used a new quantitative index, i.e., the mean pixel intensity (MPI) calculated from the pixel-intensity distribution in T1 weighted images. The corresponding MPI scores were calculated for each thigh, for each compartment and for both thighs totally (MPI_total_). The total mean pixel intensity (MPI_total_) refers to the sum of each pixel signal intensity divided by the corresponding number of pixels. An increased MPI_total_ indicates both a raised fat infiltration together with a reduced muscle volume thereby illustrating disease progression. Clinical scores did not change significantly over time whereas MPI_total_ increased significantly from an initial averaged value of 39.6 to 41.1 with a corresponding rate of 0.62/year. While clinical scores and MPI_total_ measured at the start of the study were significantly related, no correlation was found between the rate of MPI_total_ and MRC sum score changes, MFM_total_ and MFM subscores. The relative rate of MPI_total_ change was 2.3% (0.5–4.3)/year and was significantly higher than the corresponding rates measured for MRCS 0% (0–1.7) /year and MFM_total_ 0% (0–2.0) /year (p = 0.000). On the basis of these results, we suggested that muscle MRI and more particularly the MPI_total_ index could be used as a reliable biomarker and outcome measure of disease progression. In slowly progressive myopathies such as FSHD, the MPI_total_ index might reveal subclinical changes, which could not be evidenced using clinical scales over a short period of time.

## Introduction

Facioscapulohumeral muscular dystrophy (FSHD, OMIM #158900) is one of the most common hereditary muscular disorders, with a reported prevalence of 3.2–4.6 per 100,000 [1–3]. It is commonly defined as an asymmetric, descending and progressive disorder, initially affecting the face, shoulder, and arm muscles followed by the distal lower extremities and pelvic girdle [4]. Throughout the disease course, proximal lower extremities usually become involved later in time [4]. Other muscles can also be involved in the disease progression, but extra-ocular, cardiac and bulbar muscles are usually spared [4]. For the majority of FSHD patients (roughly 95%, FSHD1), the molecular basis of the pathology is a contraction of the D4Z4 macrosatellite repeat on chromosome 4q35 array from 1 to 10 repeat units (RUs) [5–7].

FSHD is a slowly progressive disorder for which the sensitivity of commonly used clinical indices to detect disease progression is still a matter of debate. On the basis of both composite manual muscle testing (MMT) and maximum voluntary isometric contraction testing (MVICT scores), a slight decline of muscle strength has been reported throughout a natural history study [8]. Statland *et al* examined data from this natural history study and from three randomized controlled trials in FSHD patients using similar techniques for strength testing [9]. On the contrary to what has been concluded from the natural history study, there was an apparent increase in strength at 6 months in 2 of the 3 clinical trials in both the placebo and treatment groups, that persisted for up to 1 year for MVICT scores. These paradoxical results illustrate that patient motivation can confound the performance measurements such as MVICT and MMT which are effort dependent. Overall, the sensitivity of commonly used indices to detect subtle changes occurring over a short period of time is poor more particularly for slowly-progressive diseases such as FSHD. Other outcome measures would be warranted for future clinical trials.

Magnetic resonance imaging (MRI) has been widely used to evaluate *in vivo* the muscle alterations invarious neuromuscular disorders including FSHD [10–12]. The corresponding quantitative MRI (qMRI) indices have not been approved yet by the European Medical Agencies as surrogate outcome measures in clinical trials [11]. In previous studies, qMRI has been used to describe the natural progression of neuromuscular disorders such as oculopharyngeal muscular dystrophy, Charcot–Marie–Tooth disease 1A, inclusion body myositis, LGMD2I and dystrophinopathies [13–19] and to characterize changes resulting from therapeutic trials [20]. Overall, it has been shown that muscle MRI can be more sensitive than clinical scores to detect disease progression during the follow-up of patients with muscular dystrophies [14,15] thereby highlighting the qMRI potential for the noninvasive assessment of disease progression and the characterization of treatment efficiency in neuromuscular disorders.

In most of the MRI studies conducted in FSHD patients, fat infiltration of the investigated muscles has been evaluated visually using the common ordinal scales including 4 to 5 grades [21–26]. In these studies muscles from upper limbs [21], lower limbs [22], both upper and lower limbs [23], or from the whole body have been investigated [24–26]. Although this qualitative scoring is of interest to determine the pattern of muscle involvement, it has been largely recognized as subjective, reader-dependent and lacking sensitivity due to the limited number of grading possibilities. Very few qMRI studies have been reported so far in FSHD patients [27–31]. In these studies, a significant correlation between intramuscular fat fraction and severity scores and inverse correlations with muscle strength have been reported [27–29]. Given that quantitative approaches are not operator-dependent, one can hypothesize that qMRI might be more accurate than visual scores for the evaluation of disease progression and effectiveness of any therapeutic intervention. Using a fully automatic segmentation method of MR images, we have previously quantified in FSHD patients fatty infiltration in both thighs [30]. Interestingly, the quantified intramuscular fat fraction was significantly correlated to the visual and the clinical scores. However, for patients with a severe fat infiltration i.e. a score ≥3, the corresponding quantitative intramuscular fat fraction was within a very large range suggesting a ceiling effect of the visual score and a potentially larger sensitivity of qMRI to muscle fat infiltration. In our previous study, we did not characterize the corresponding time-dependent changes which could be of interest if such an approach is intended to be used for the characterization of therapeutic interventions.

The main aim of the present study was to investigate longitudinally the time-dependent MRI changes occurring in thigh muscles of FSHD patients and to assess the potential relationships with clinical findings.

## Patients and methods

### Subjects

Thirty-five FSHD patients were enrolled in this study after providing a written informed consent. They were part of the outpatients seen at the neuromuscular diseases and ALS reference center at CHU la Timone, Marseille between November 2010 and July 2013. For each patient, data related to age, sex, body mass index (BMI), age at onset, first symptoms, duration of the disease and size of the contracted D4Z4 allele expressed as the number of RUs were recorded. Patients were included if they had a typical clinical phenotype consistent with FSHD and harbored a 4qA contracted allele with an estimated size lower than 10 RUs (40 kb).

Subjects with at least one of the following criteria were excluded: wheelchair-bound patients, patients with concomitant diseases that may cause myopathic or neurogenic findings on MRI, non-penetrant carriers of a contracted D4Z4 array, and patients with a typical clinical phenotype of FSHD without a contracted D4Z4 array (FSHD2).

Fifteen age-matched healthy volunteers were enrolled as controls from the local population by poster advertisements in the Neurology department. The protocol was approved by the local ethics committee (Comité de Protection des Personnes Sud Méditerranée I).

### Clinical assessment tools

#### Manual muscle testing

Manual muscle testing (MMT) was performed using a modified Medical Research Council (MRC) quantitative scale including five levels (from 0: no movement to 5: normal movement). Quadriceps and hamstrings (on each side) were evaluated. In each thigh, the scores of knee flexion and extension were added as right and left thigh MRC score (range: 0–10), and the sum of scores of both thighs was defined as the MRC sum score (MRCS) (range: 0–20). Clinical muscle function assessment was not performed on the healthy volunteers.

#### Clinical Severity Score (CSS)

Disease severity was evaluated using the Clinical Severity Score (CSS) scale [32]. The score ranges from 0: no deficit to 5: wheelchair-bound.

#### Motor function measurement (MFM)

The MFM scale was used as previously described [33]. It could be divided into three dimensions: D1 for standing position and transfer, D2 for axial and proximal limb motor function and D3 for distal motor function and total MFM sum score (MFM_total)_. For the description of each subscale as well as MFM_total_, each score was expressed in percent of the maximal value.

### Follow-up period

The patients were followed for a period ranging from 1 to 2 years with a visit every year. Both thighs were imaged as previously described [30]. For each MRI session at the same time, a complete neurological exam including MMT and MFM was also performed. The same neurologist (ESC) performed baseline and follow-up clinical examinations in each patient

### Muscle MRI

Muscle MRI was performed using an Avanto 1.5T MR scanner (Siemens, Erlangen-Germany). Both thighs were imaged using a flexible coil on the top and a spine coil on the bottom. T1-weighted (T1W) images in the axial plane (from 35 to 50 slices according to the height of the patient) were recorded with the subsequent parameters (400 mm field of view, 160 * 320 acquisition matrix, 4 mm slice thickness, 2 mm gap). The TR-TE values (ms) were 578–11, the flip angle was 90° and the refocusing flip angle was 120°. The total acquisition duration was 2.21 minutes. Image uniformity correction (pre-scan normalization) was used to reduce signal inhomogeneities due to the receive coils.

#### Image processing

**Fig 1:**
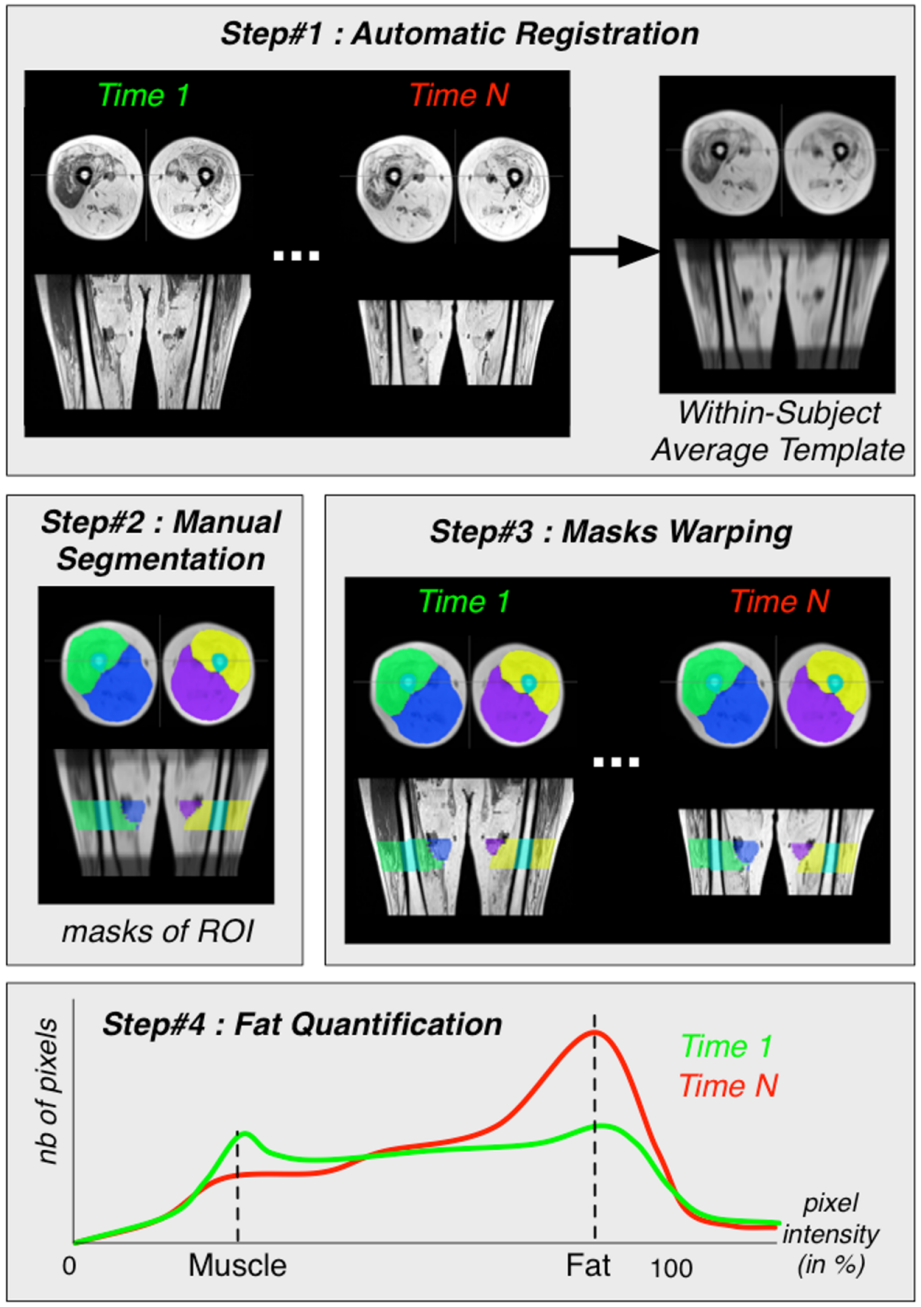
Pipeline of the four-step image processing.

A four-step processing pipeline was used including, as illustrated in Fig 1, an initial registration process (step#1), a manual segmentation (step#2), a warping process (step#3) and a normalization procedure of histograms (step#4).

Step #1: The registration process was similar to what we previously reported [34]. For each subject, an averaged T1-weighted (T1w) template was created using the whole set of T1w images recorded over time. Each image was initially corrected for any remaining signal inhomogeneities using the N4 algorithm [35] and then registered to the initial T1w image as previously described [36–38].
Step #2: For each averaged template, masks of interest i.e. bone, anterior and posterior compartments were carefully manually drawn by the same observer (FF) using FSLview software [39]. We took care of not selecting areas from the subcutaneous fat compartment.
Step#3: For each subject, the masks were warped to each original T1w images using the inverse deformation fields estimated during the initial non-linear registration process as previously described [36–38].
Step #4: For each region of interest, the pixel intensity distributions (histograms) were normalized with a linear interpolation using the bone marrow intensity in the lumen of the femur as the 100% reference.

Step#1 illustrates the creation of the average template (right-side) using the initial T1W images (left hand-side). Step#2 illustrates the manual segmentation of each compartment (anterior, posterior and bone). Step#3 illustrates the masks warping to the original T1W images. Step#4 illustrates the MPI_total_ quantification from the normalized histogram.

#### MRI indices

**Fig 2:**
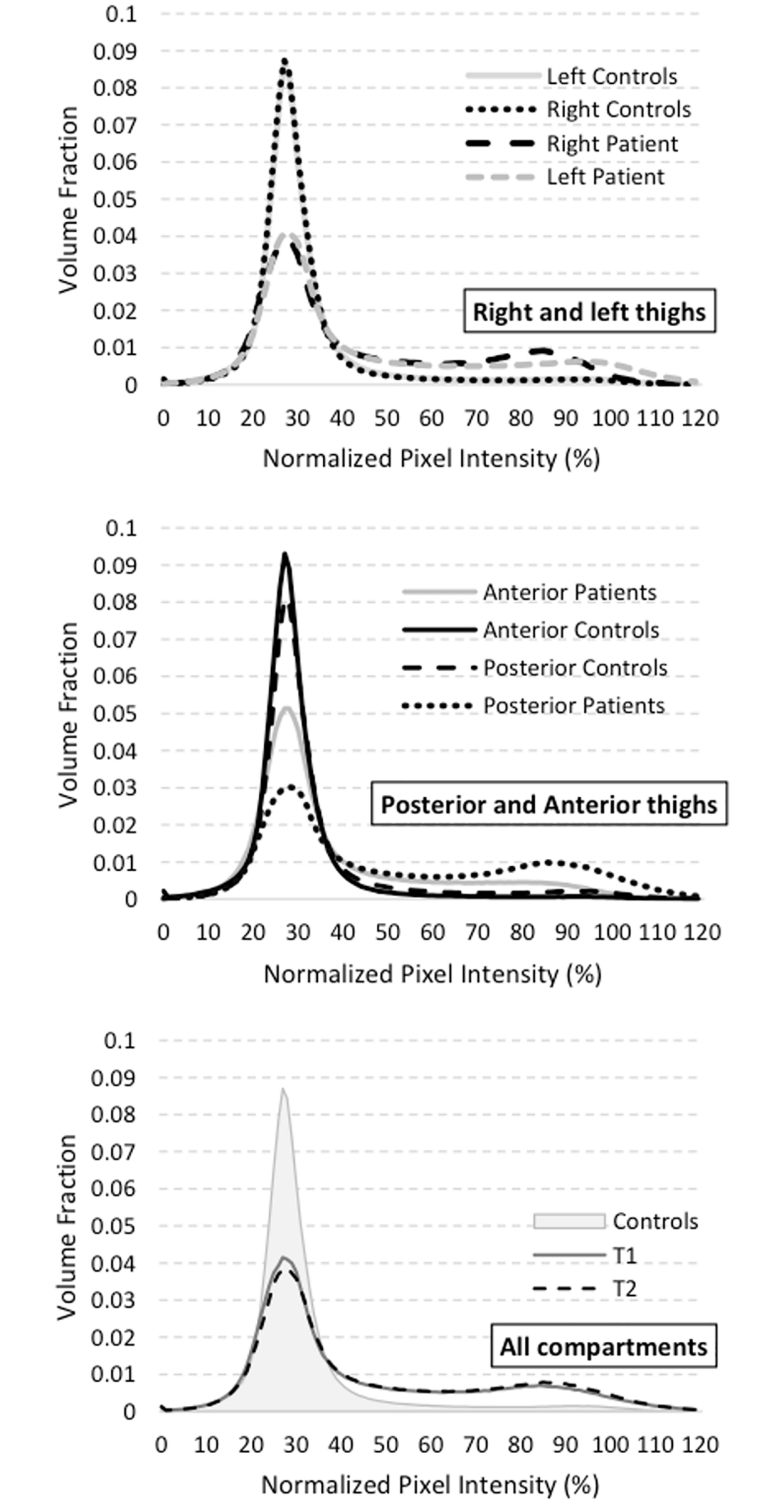
Normalized histogram showing the normalized pixel intensity distribution computed at two different time points (T1 and T2) and comparing volume fraction on left/right thighs and anterior/posterior compartment of thighs between controls and patients.

The normalized histogram shown in Fig 2, illustrates the pixel intensity distribution i.e. the relative number of pixels (volume fraction %) with respect to the normalized pixel intensity, in the corresponding T1W image and was used to generate an MRI index. The total mean pixel intensity (MPI_total_) refers to the total signal intensity (i.e. the sum of each pixel signal intensity) divided by the corresponding number of pixels. MPI_total_ takes into account the bimodal pixel intensity distribution with one mode on the left-hand side illustrating the pixel intensities corresponding to muscle and a second mode on the right-hand side representing the pixel intensities corresponding to fat. An MPI_total_ increase indicates both a raised fat infiltration together with a reduced muscle volume thereby illustrating disease progression. The MPI_total_ index was quantified for each thigh and each compartment.

The normalized pixel intensity distribution is bimodal with one mode on the left-hand side illustrating the pixel intensities corresponding to muscle and a second mode on the right-hand side illustrating the pixel intensities corresponding to fat. The bottom figure shows the normalized pixel intensity distribution of both thighs for controls comparing with patients at T1 and T2. The figure in the middle shows the normalized pixel intensity distribution of anterior and posterior compartments of thighs for patients and controls. The upper figure shows the normalized pixel intensity distribution of right and left side of patients and controls.

#### MPI_total_ reproducibility

In order to assess MPItotal reproducibility, two muscle MRI were performed on two separate days in 7 healthy volunteers and measurements of MPI were repeated on both.

### Rates of changes

For each variable, a rate of change was quantified as the difference between two time points divided by the time between the corresponding time points (in years).

In order to compare the rate of changes in the different clinical and MRI variables, we calculated relative rates of changes as follows: [100 × (Score at last visit–Score at T1) / Score at T1]/total duration between last visit and T1 (in years).

### Statistical analysis

Data analysis was performed using RStudio (R version 3.2.2). Considering the non-gaussian distribution of the variables, repeated measure analyses were performed using the Wilcoxon rank test for measurements repeated twice and Friedman test for measurements repeated more than twice. We reported the v and the F values for the Wilcoxon and Friedman tests respectively. The v values correspond to the sum of ranks assigned to the differences with a positive sign. Spearman correlation analysis was used to calculate the correlation between clinical scores and MRI parameters. To compare relative rates of changes between scores, we used Kruskal–Wallis test and performed post-hoc analysis with Wilcoxon test and Bonferroni correction.

For the whole set of statistical tests, the significance level was set at p<0.05.

Repeated MPI_total_ measurements were assessed using a paired t test and the corresponding reproducibility was determined as the mean absolute interscan differences (M). The limit of agreement for a 95% level of confidence was calculated according to Bland and Altman as M ± 1.96 SD [40]. The intra-class correlation coefficient (ICC) between the repeated measurements was calculated as previously described [41] and used to assess the relative reliability of the MPI variable.

Considering the data distribution, results are presented as median [interquartile range (IQR: 25^th^ –75^th^ percentiles)].

## Results

### Clinical characteristics of the patients

In this study, 35 patients (17 female) were enrolled. Their age at the start of the study was 45 (36–56) years and was 27.5 (16–41) years at the onset of the first symptoms. All of them had a clinical and a radiological exam at two time periods (T1 and T2). For 11 patients, an additional session including MRI and clinical examination (3 exams) was performed (T3). At the start of the study, patients BMI was 23.9 (21.6–26.1) kg/m^2^ and the median CSS score was 3.0 (2.5–3.0). The median time between T2 and T1 was 12.5 (12–15.5) months and between T3 and T2 was 13.5 (12–20.5) months. BMI did not change over time with median values at T2 and T3 being 24.2 (20.9–26.9) and 22.8 (22.1–25.85) kg/m^2^.

### Change over time of standardized neurological scores

#### MMT

**Fig 3:**
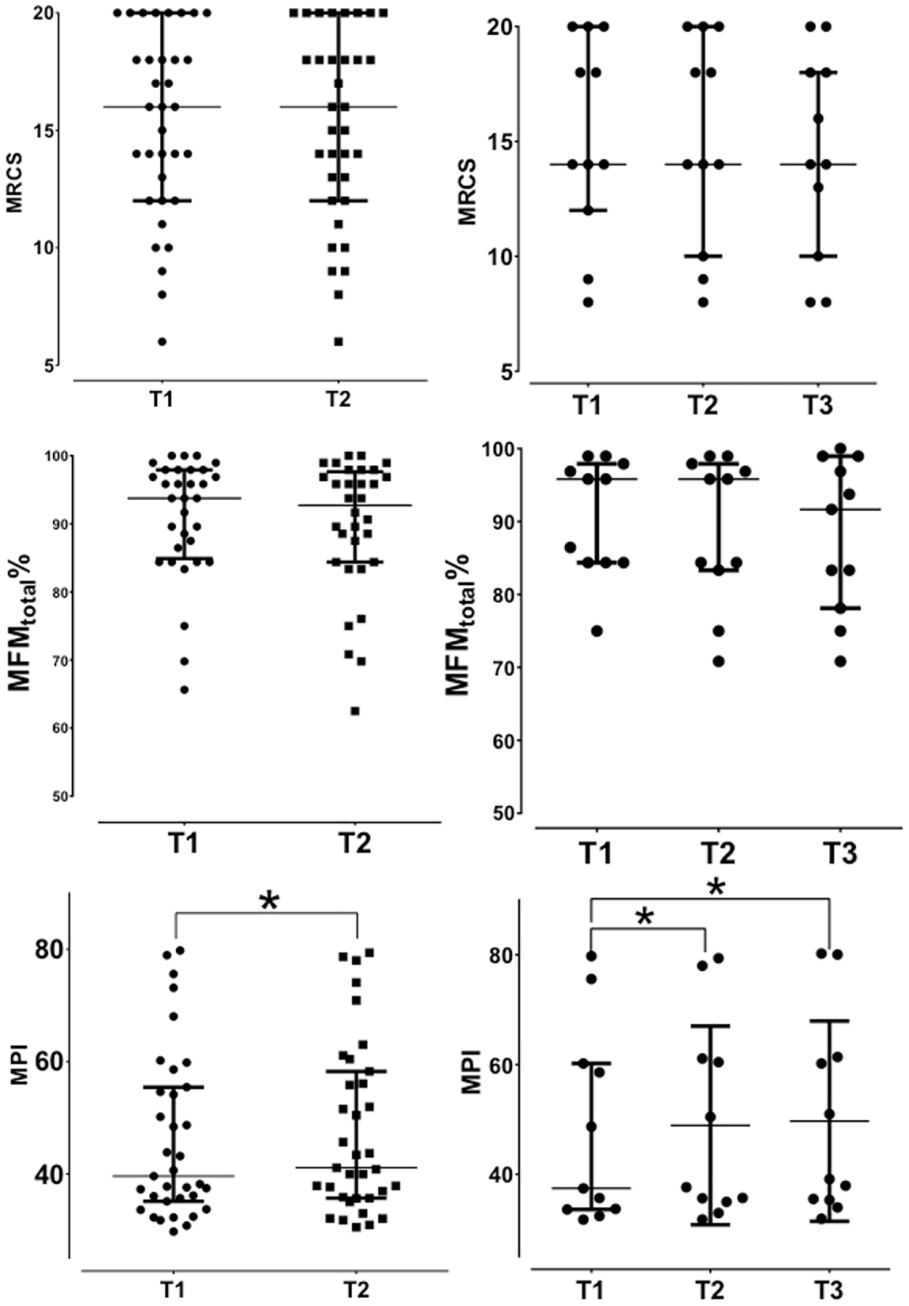
Progression of disease as measured by time-dependent changes in MRCS, MFM total) and MPI. Results [median (interquartile range)] are presented for all patients (n = 35) at T1 and T2 (left side) and for patients (n = 11) who had 3 visits (T1, T2, T3) (right side). The median time between T2 and T1 was 12.5 months (12–15.5) and between T3 and T2 was 13.5 months (12–20.5). The symbol * indicates significant change. MPI: mean pixel intensity; MRCS: MRC sumscore; MFM: motor function measurement.

As illustrated in Fig 3, at the start of the study, MRCS was 16.0 (12.3–18) and remained unchanged at T2 16.0 (12.0–18.0) (v = 23.5, p = 0.46). Repeated measures for the patients with 3 exams showed no statistically significant change in manual muscle testing in both thighs at T1 16.0 (11.3–18.5), T2 16.0 (9.8–18.5), and T3 16.0 (9.5–18.5) [F (2, 16) = 2, p = 0.17] (Fig 3). The annual rate of MRCS change between T2 and T1 was 0 (0–0) and was identical (-0.35–0) between T3 and T2 and between the last exam and the first (-0.03–0).

#### MFM

The MFM D1% median value did not change with respect to time with T1 value being 87.2 (67.9–94.9) % and T2 value being 87.2 (71.2–94.9) % (v = 40.5, p = 0.20). Likewise, for patients with 3 exams, the Friedman test indicated a stability over time with the D1 value at T1 being 89.7 (66.7–96.8) %, at T2 89.7 (65.4–96.8) %, and at T3 87.2 (61.5–96.8) % [F (2, 20) = 0.81, p = 0.45] (Fig 3). The annual rate of change between T2 and T1 was 0 (0–0) %, and was identical 0 (-3.0–0) % between T3 and T2, and between the last and the first exam was 0 (-0.01–0) %.

The MFM D2% at the start of the study was 100 (97.2–100) % and was unchanged at T2 100 (97.2–100) % (v = 10, p = 0.13). For patients with 3 exams, Friedman test revealed no statistically significant change in MFM D2% in both thighs at T1 100.0 (97.2–100.0) %, T2 100.0 (97.2–100.0) %, and T3 97.2 (97.2–100) % [F (2, 20) = 0.81, p = 0.09] (Fig 3). The annual rate of change between T2 and T1 was 0 (0–0) %. Accordingly, the annual rate of MFM D2% change was 0 (0–0) %.

The initial MFM D3% score was 100 (95.2–100) % and the value measured at T2 [100 (95.2–100) % (v = 15, p = 0.06)] was not statistically different. Repeated measured over time did not illustrate statistically significant change with values at T1 being 100.0 (100.0–100.0) %, at T2 100.0 (96.4–100.0) %, and at T3 100.0 (95.2–100.0) % [F (2, 20) = 0.73, p = 0.49] (Fig 3). The annual rate of change was 0 (0–0) % between T2 and T1, and between T3 and T2 was 0 (-0.9–0) %. Accordingly, the annual rate of MFM D3% change between the last and the first exams was 0 (0–0) %.

The MFM_total_% score at T1 was 93.8 (85.9–97.9) % and did not change at T2 [93.8 (84.4–97.1) % (V = 51.5, p = 0.10)]. Similarly, for patients with 3 exams, Friedman test revealed a non-significant increment of MFM_total_% at T1 95.8 (84.9–97.9) %, T2 95.8 (84.4–97.9) %, and T3 93.8 (83.3–98.7) % [F (2, 20) = 0.1.08, p = 0.36] (Fig 3). The annual rate of change was 0 (-0.4–0) % between T2, and T1 and -0.2 (-1.3–0) between T3 and T2. The MFM_total_% rate of change between the last and the first exam was 0 (-1–0) %.

### Reproducibility of MPItotal measurements

MPI_total_ values measured repeatedly 2 days apart in 7 healthy volunteers were 30 ±2.9 and 29.9 ± 2.9 respectively. The mean absolute difference was 0.28 ± 0.20, the corresponding ICC was 0.99 and the limits of agreement ranged between -0.1 and 0.68.

### MPI_total_ change over time

The time-dependent changes in MPI_total_ are reported in Fig 3, and the corresponding values for each compartment are summarized in Table 1. As illustrated in Fig 2, the pixel intensities distribution slightly shifted downwards in the left part and upwards in the right part thereby indicating a muscle loss and an increasing fat infiltration over time.

**Table 1:** MPI_total_ values in different compartments at different time periods.

Thigh Compartment	T1	T2	T3	p	p1,2	p2,3	p1,3
**Right Anterior**	34.66 (30.73–47.42)	37 (32.02–52.87)		**0.000**	*		
	33.02 (30.21–54.97)	34.21 (31.05–57.98)	34.19 (30.93–47.76)	**0.**086			
**Right Posterior**	45.96 (36.27–59.21)	47.31 (37.35–60.03)		**0.005**	*		
	41.16 (36.27–71.57)	40.85 (36.2–73.85)	42.85 (36.98–73.21)	0.052			*
**Left Anterior**	31.37 (28.57–42.86)	32.8 (29.34–44.5)		**0.000**	*		
	30.12 (28.4–62.86)	30.22 (29.05–62.68)	32.31 (28.6–53.37)	**0.029**		*	*
**Left Posterior**	48.44 (40.48–69.68)	50.77 (43.04–69.78)		**0.003**	*		
	46.19 (40.48–83.18)	46.55 (40.25–83.26)	49.27 (41.22–84.54)	**0.002**		*	*
**Anterior**	32.51 (29.77–43.99)	35.32 (30.9–44.9)		**0.000**	*		
	31.6 (29.29–58.83)	31.66 (30.66–60.33)	33.19 (29.41–47.79)	**0.020**	*		*
**Posterior**	45.99 (39.15–62.03)	45.99 (39.65–64.64)		**0.001**	*		
	43.5 (37.96–80.91)	43.49 (39.51–81.24)	45.81 (40.17–81.81)	**0.006**			*
**Right**	39.31 (34.15–55.34)	41.24 (35.21–56.83)		**0.000**	*		
	36.7 (33.49–57.51)	36.61 (34.35–58.21)	37.84 (34.2–58.58)	**0.026**	*		*
**Left**	40.04 (34.31–58.43)	42.16 (36.16–57.31)		**0.000**	*		
	38.27 (33.91–63.4)	39.09 (34.7–64.35)	40.61 (34.31–66.35)	**0.012**			*
**Both thighs**	39.58 (35.11–55.44)	41.08 (35.68–58.25)		**0.000**	*		
	37.44 (33.6–60.2)	37.65 (34.99–61.09)	39.13 (35.3–61.38)	**0.004**	*		*

MPI_total_: mean pixel intensity. T1 refers to baseline, T2 to 12.5 months (12–15.5) after T1 and T3 to 13.5 months (12–20.5) after T2. For patients with 3 exams, post-hoc analysis with Bonferroni correction has been performed.

The symbol * indicates significant change between two time points (p1,2: p between T1 and T2; p2,3: p between T2 and T3; p1,3: p between T1 and T3).

As shown in both Table 1 and Fig 3, a significant increase was found for MPI_total_ with values ranging from 35.1 to 55.4 at the start of the study and from 35.7 to 58.2 at T2 (v = 55, p = 0.000). For patients with three repeated exams, Friedman test revealed a significant MPI_total_ increase with values ranging from 33.6 to 60.2 at T1, from 34.9 to 61.1 at T2 and from 35.3 to 61.38 at T3 [F (2, 24) = 7.54, p = 0.004]. The rate of MPI_total_ change (/year) was 0.62 (0.01–1.79)/year between T1 and T2, 0.30 (0.08–0.95)/year between T2 and T3, and 0.63 (0.01–1.79)/year between the last exam and the first exam.

The rates of MPI_total_ change were 1.1 (0.09–2.01)/year for the anterior compartment, 1.73 (-0.06–2.31)/year for the posterior compartment (without significant difference between the anterior and posterior compartments, p = 0.48), 0.86 (-0.04–2.06)/year for the right thigh and 1.75 (0.00–1.93)/year for the left thigh with no significant difference between the different sides (p = 0.40). The rate of total MPI_total_ change was 1.27 (0–1.85)/year.

According to the CSS scores measured at T1, we pooled the patients in two groups with 12 patients with a CSS score < 3 and 23 patients with a CSS score ≥ 3. The rate of MPI_total_ change was similar in both groups [CSS < 3: 0.42 (0.05–1.72)/year and CSS score ≥ 3: 1.22 (0–1.95)/year; p = 0.37]. The rates were also identical for the anterior [CSS < 3: 0.43 (0.05–1.09)/year and CSS score ≥ 3: 1.30 (0.39–2.11)/year; p = 0.07] and posterior compartments [CSS < 3: 0.47 (0.20–2.10)/year and CSS score ≥ 3: 1.02 (0.15–2.34)/year; p = 0.07].

### Correlation between MRI quantitative indices and clinical scores

The correlation coefficients between the clinical scores including MRC, CSS, and MFM D1%, MFM D2%, MFM D3%, MFM_total_ % and MPI_total_ are reported in Table 2 for each session. At T1, we found a significant correlation between the duration of the disease and the MPI_total_ (r = 0.37, p = 0.030). On the contrary the disease duration was linked neither to MRCS (r = -0.14, p = 0.42), CSS (r = 0.19, p = 0.26), MFM D1% (r = -0.07, p 0.70), MFM D2% (r = -0.32, p = 0.70), MFM D3% (r = 0.04, p = 0.82) nor to MFM_total_ % (r = -0.06, p = 0.76). The negative relationship between the MPI_total_ and the number of RUs was not statistically significant (r = -0.22, p = 0.22). We did not find any relationship between MPI_total_ and BMI at any time during the follow-up period (T1: r = 0.15, p = 0.41; T 2: r = 0.22, p = 0.22; T 3: r = 0.06, p = 0.85).

**Table 2:** The correlation between clinical scores and MPI_total_ at the start of the study.

	T1	p
Right thigh MRC**	**-0.63**	**0.000**
**Left thigh MRC**	**-0.68**	**0.000**
MRCS***	**-0.66**	**0.000**
**CSS**	**0.65**	**0.000**
**MFM**†**D1%**	**-0.64**	**0.000**
**MFM D2%**	**-0.37**	**0.037**
**MFM D3%**	**-0.22**	**0.228**
MFM_total_	**-0.62**	**0.000**

* MPI_total_: mean pixel intensity,

**MRC: Medical research council,

***MRCS: MRC sumscore,

^†^MFM: motor function measure. T1 refers to baseline measurement.

### Correlation between the rates of MRI indices changes and the clinical scores changes

The inverse relationship between the duration of the disease and the rate of MPI_**total**_ change was not statistically significant (r = -0.21, p = 0.23). Also, no significant correlation was identified between the age of patients at T1 and the rate of MPI_**total**_ change (r = 0.12, p = 0.48).

We analyzed the potential correlations between the rate of change for each clinical score and the corresponding rate of MPI_total_ change. As indicated in Table 3, none of the clinical score rate of change was correlated with the rate of MPI_total_ change over time. We did either find no correlation between the MPI and BMI rates of changes (Time 1,2 and MPI 1,2: r = 0.30, p = 0.10, Time 2,3 and MPI 2,3 (n = 11): r = -0.18, p = 0.64).

**Table 3:** Correlation between the rate of MPI_total_* change and the rate of clinical scores change.

Scale	r	p
MRCS**	0.04	0.81
**MFM**† **D1%**	-0.17	0.34
**MFM D2%**	0.22	0.23
**MFM D3%**	-0.07	0.70
**MFM**_**total**_**%**	-0.06	0.76

*MPI_total_: mean pixel intensity,

**MRCS: MRC (medical research council) sumscore,

^†^MFM: motor function measure

No correlation was found between the rate of MPI_total_ and MRCS changes (r = 0.04, p = 0.81), MFM D1% (r = -0.17, p = 0.34), MFM D2% (r = 0.22, p = 0.23) MFM D3% (r = -0.07, p = 0.70) and MFM_total_% (r = -0.06, p = 0.76). Similarly, the rate of MPI_total_ change was not significant correlated to the number of repetition units (r = 0.05, p = 0.80).

### Relative rates of changes

**Fig 4:**
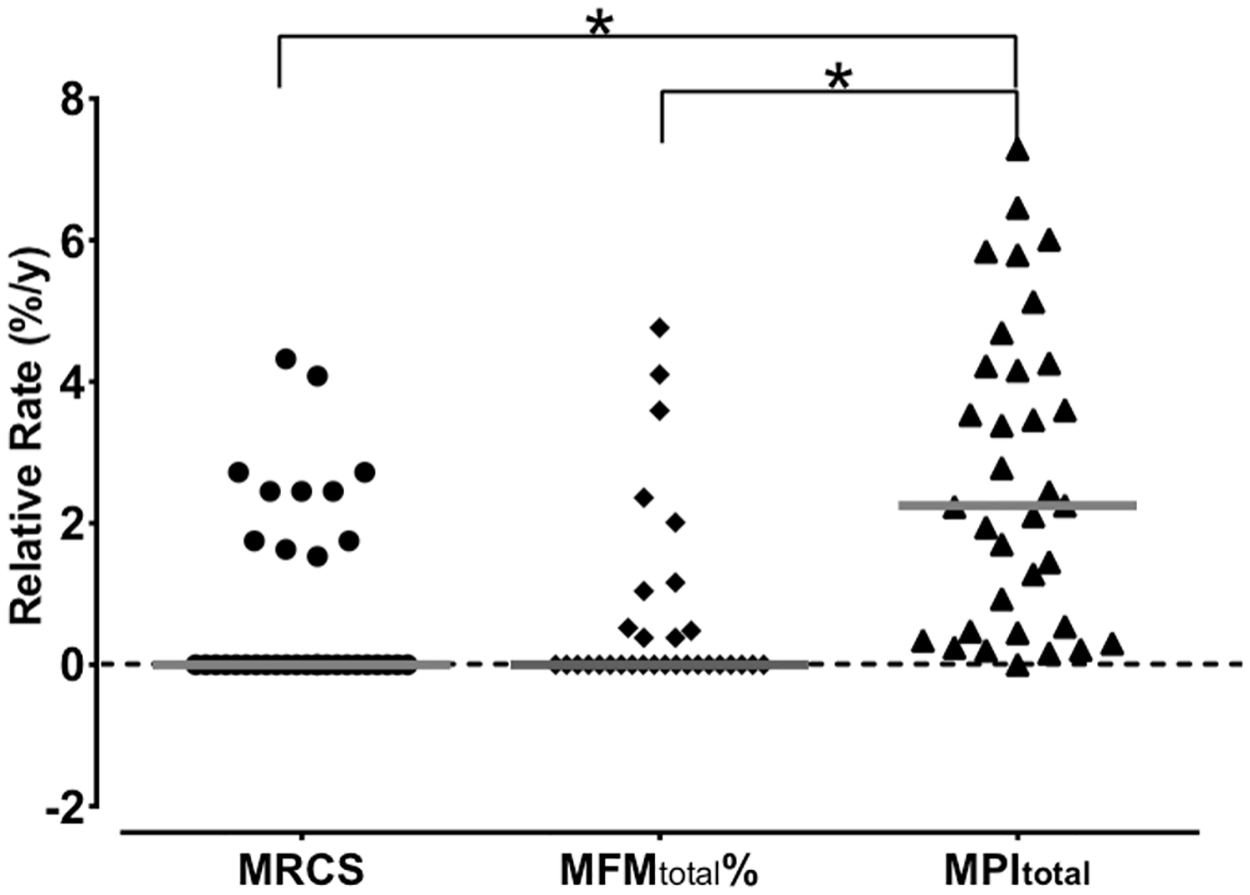
Relative rates of changes for the different clinical and MRI variables. MFM_total_: mean pixel intensity, MRCS: MRC sumscore, MFM: motor function measurement. The symbol * indicates significant difference.

The relative rate of MPI_total_ change was 2.3 (0.5–4.3) %/year and was significantly higher than the corresponding rates measured for MRCS 0 (0–1.7) %/year and MFM_total_ 0 (0–2.0) %/year (×^2^ = 23.6, p = 0.000) (Fig 4).

## Discussion

In this study, we longitudinally investigated muscle changes in FSHD patients using traditional clinical scores and a new MRI index i.e. MPI which is a quantitative index calculated from the pixel intensity distribution in T1W images and illustrating both muscle loss and fat infiltration in thigh muscles. We aimed at characterizing the natural history of the disease and at investigating the potential correlations between the rate of clinical and MRI changes.

Considering the natural history, FSHD is primarily recognized as a slowly progressive disorder with slight changes of clinical scores over a short period of time. An average MMT decline of 0.07/year has been reported and was not associated with either age, age at onset, gender, or disease duration.[8]. This slow disease progression and the poor sensitivity of the commonly used clinical scores mandates the utilization of more sensitive biomarkers which could be used to appropriately assess disease progression and the potential effects of therapeutic interventions.

In this study, we observed a strong correlation cross-sectionally between clinical scores and MRI indices at baseline and follow-up. This is consistent with previous cross-sectional studies in FSHD [27–29] and in other MRI studies in muscular dystrophies [14,15]. This relationship was not found for the longitudinal measurements. Interestingly, MPI_total_ significantly changed over the reported period in all the muscle compartments whereas the clinical scores (MFM, CSS, MRCS) did not. These results indicated that MPI_total_ would be more sensitive to mild changes over a short period of time. A similar conclusion has been reported for other slowly progressive diseases [15,18]. In a natural history study of patients with LGMD2I, a significant increase in muscle fat fraction has been reported throughout a one-year follow-up whereas no changes were reported for any of the conventional longitudinal physical assessments [15]. Accordingly, in a small group of patients with Becker Muscular Dystrophy [18], the rate of fat fraction change was 3.7%/year whereas the clinical changes measured by the MFM were relatively mild, with a decrease of around 1%/year. In a recent one-year longitudinal study of patients with Duchenne muscular dystrophy (DMD), Bonati *et al*. reported a significant increment of the mean fat fraction in all the patients and all studied thigh muscle groups [17], a significant decrease of mean MFM_total_ score (2.9%/year) and a highly negative correlation between clinical scores (MFM_total_ and D1 subscore) and thigh mean fat fraction values longitudinally (r = −0.71).

In our cohort, we observed a significant MPI_total_ increase with a 2.3% (0.5–4.3)/year relative rate of change. In a recent study conducted in FSHD patients following a 16-week training period, Janssen *et al*. [31] used quantitative T2 MRI in order to quantify the rate of fat infiltration progression. They reported that fat infiltration significantly decreased in patients receiving aerobic exercise training (2.9%/year) or cognitive-behavioral therapy (1.7%/year) whereas fat fraction increased (6.7%/year) in patients receiving usual care. The apparent discrepancy between the rate of MPI_total_ change reported in our study and the rate of fat fraction change reported by Janssen *et al*. could be due to methodological differences. The fat fraction reported in the study of Janssen *et al* was quantified using a biexponential fitting of multiecho T2W images whereas we quantified MPI_total_ from the histogram analysis of T1W images. In addition, one has to keep in mind that MPI not only accounted for fat infiltration but also to the corresponding muscle loss which is not directly quantified using the T2 fitting method.

From a methodological point of view, the MPI_total_ calculation did not display any operator dependency. In a previous study [30], the index of fat infiltration of the muscle compartment (IFI) we used as a quantitative surrogate of fat infiltration required the determination of a threshold in order to distinguish muscle and fat compartments. In contrast the MPI_total_ calculation does not depend on a threshold as it takes into account the entire range of pixel intensity distribution. After manual compartment delineation, the process of MPItotal calculation is automated. It is also noteworthy that we have measured IFI in the same group and IFI was strongly correlated to MPI.

In order to assess the reliability of the MPI_total_ metric, we performed a test/retest procedure in healthy controls. The repeated MPI measurements performed over a period of 2 days were highly similar, demonstrating a good reproducibility. The ICC between the repeated measurements was 0.99 and the limits of agreement ranged between -0.1 and 0.68. The limits of agreement illustrate the sensitivity to detect meaningful change. For instance, the upper limits of agreement indicated that 0.68 MPI_total_ unit would be a significant change that could be detected for a given subject at the 95% confidence level. The absolute value is similar to what has been previously reported for fat fractions quantified using Dixon techniques i.e. around 1 [42] and is by far lower than the 2.3%/year average rate of change we reported in the present study in patients thereby illustrating the sensitivity of MPI metric to detect significant changes in FSHD patients

We have to acknowledge some limitations related to this study. We assessed muscle loss and fat infiltration based on T1W images as previously described [30] rather than a chemical-shift based method. The Dixon techniques using more than 3 echo times would be expected to handle biasing factors such as B0 field inhomogeneity, T2 relaxation, phase and the spectral complexity of fat, which permits measurement of the PDFF (proton density fat fraction) in the tissues. In that respect, MPI would be expected not to have the potential accuracy of Dixon methods to assess fat infiltration. However, T1W imaging is also not susceptible to the same confounders since it depends only on the difference in T1 between fat and muscle. The principal challenge is that signal intensity is also a function of the transmit and receive coils as well as T1. As we used a spin echo sequence with a 90° flip angle, we could tolerate some B1 inhomogeneity, in that variation of up to 20% in the flip angle should lead to less than 5% changes in signal. The signal inhomogeneity due to the coil profile can be also mitigated by using image uniformity corrections intended to minimize this type of bias (e.g. pre-scan normalization and N4 post-processing). Considering the dependence of T1 with respect to the MR field strength, MPI values might only be comparable as long as the measurements are performed using the same field strength. This would be a limitation for multicenter trials. Another limitation is that we normalized the pixel intensity distribution with respect to the bone marrow signal in the lumen of the femur. This normalization procedure would not be suitable for pediatric populations where red marrow may be present in the mid-femur region.

Overall, although it might be an advantage to use PDFF as an index of fatty infiltration, our normalized T1W-derived measure of fat content was already more sensitive to disease progression than the current clinical standard scoring. In addition, considering that the qMRI analysis has been performed similarly for the whole set of subjects, one can consider that the potential bias was the same in each case and did not compromise the reliability of the results. Moreover, the repeated measurements performed two days apart illustrated the high reproducibility of the MPI metric.

In conclusion, in the present study, we showed that qMRI and more particularly the MPI_total_ index may be useful for the assessment of the disease progression in FSHD. Muscle qMRI may reveal muscle changes which cannot be evidenced using clinical scales and therefore may provide reliable and non-invasive outcome measures for clinical trials.

## Supporting information

S1 Table Summary of individual genetic data, BMI, sex and muscle scores across all FSHD patients. (PDF)

S2 Table Summary of MPI_total_ for all FSHD patients. (PDF)

S1 Fig Three examples of manual segmentation. S1 Fig shows three examples of T1-weighted (T1W) images in the axial plane at T1, T2, T3 with manual segmentation of anterior and posterior compartment of thighs (Patients P1, P7 and P19).
(WEBARCHIVE)
