# Supplementary material for: Long-term follow-up of MRI changes in thigh muscles of patients with Facioscapulohumeral dystrophy: A quantitative study
Source: PLoS One. 2017 Aug 25;12(8):e0183825. doi: 10.1371/journal.pone.0183825 (PMC5571945; doi:10.1371/journal.pone.0183825)
Supplement: S1 Table — (PDF) [file pone.0183825.s001.pdf]

Table S1. Demographic and clinical features

Code	Age at MRI 1	Sex	Age at onset	RUs	MRC total T1	MRC total T2	MRC total T3	CSS T1	MFMD1 T1	MFMD2 T1	MFMD3 T1	MFMtotal T1	MFMD1 T2	MFMD2 T2	MFMD3 T2	MFMtotal T2	MFMD1 T3	MFMD2 T3	MFMD3 T3	MFMtotal T3	BMI
**P1**	44	M	15.5	5	9	8	8	3	74.35897	97.22222	90.47619	86.45833	69.23077	97.22222	90.47619	84.375	53.84615	97.22222	90.47619	78.125	26.89232
**P3**	36	F	18	5	20	20	20	1.5	97.4359	97.22222	100	97.91667	97.4359	97.22222	100	97.91667	100	97.22222	100	98.95833	21.35991
**P4**	69	M	55	7	18	18	0	2.5	92.30769	100	95.2381	95.83333	92.30769	100	95.2381	95.83333					20.76125
**P7**	42	M	32	6	14	15	14	3	89.74359	100	100	95.83333	89.74359	100	100	95.83333	89.74359	97.22222	95.2381	93.75	19.44444
**P8**	49	F	42	7	18	18	16	3	64.10256	100	95.2381	84.375	61.53846	100	95.2381	83.33333	61.53846	100	95.2381	83.33333	21.64412
**P10**	19	M	17	8	20	20	0	1.5	100	100	100	100	100	97.22222	100	98.95833					32.31434
**P11**	44	M	35	11	20	20	0	3	82.05128	100	95.2381	91.66667	82.05128	100	95.2381	91.66667					23.91327
**P12**	55	F	42	5	12	12	0	4	64.10256	97.22222	100	84.375	43.58974	97.22222	100	76.04167					25.35154
**P14**	69	F		8	20	20	0	1.5	94.87179	97.22222	100	96.875	94.87179	97.22222	100	96.875					25.28257
**P15**	39	M	14	5	14	14	14	3.5	61.53846	100	100	84.375	64.10256	97.22222	100	84.375	61.53846	97.22222	100	83.33333	19.23018
**P16**	52	M	41	8	15	15	0	2.5	87.17949	97.22222	100	93.75	87.17949	97.22222	100	93.75					18.37683
**P17**	42	F	16	5	16	18	0	1.5	100	100	100	100	100	100	100	100					20.02884
**P18**	51	F	15	6	18	18	0	3.5	76.92308	97.22222	100	89.58333	76.92308	97.22222	100	89.58333					33.26214
**P19**	48	F	38	8	14	14	13	3	92.30769	100	100	96.875	92.30769	100	100	96.875					25.52964
**P20**	63	F	35	7	8	9	8	4.5	43.58974	97.22222	95.2381	75	43.58974	97.22222	95.2381	75					23.82813
**P21**	70	F	41	7	11	11		4.5	28.20513	100	95.2381	69.79167	28.20513	100	95.2381	69.79167					28.22839
**p23**	56	M	51	8	20	20	20	2.5	89.74359	100	100	95.83333	89.74359	100	100	95.83333	82.05128	100	95.2381	91.66667	
**P25**	56	M	51	8	14	14		3	87.17949	100	95.2381	93.75	79.48718	100	90.47619	89.58333					28.02482
**P26**	26	F	13.5	7	17	14		3	97.4359	97.22222	100	97.91667	74.35897	88.88889	90.47619	83.33333					20.0796
**P27**	40	F	28	7	13	13		3	76.92308	97.22222	100	89.58333	76.92308	97.22222	95.2381	88.54167					24.02381
**P28**	49	F	19	6	12	12		3.5	71.79487	100	100	88.54167	71.79487	100	100	88.54167					25.5102
**P29**	28	M	16	5	10	9		3.5	89.74359	100	100	95.83333	89.74359	100	100	95.83333					18.14487
**P30**	23	M	11	5	14	13		3													24.81096
**P31**	60	M	49	5	18	18		2.5	79.48718	94.44444	90.47619	87.5	79.48718	94.44444	90.47619	87.5					28.39373
**P32**	50	F	27	7	20	20	18	3	97.4359	100	100	98.95833	97.4359	100	100	98.95833	97.4359	100	100	100	
**P33**	36	M	19	9	18	18	18	1.5	100	97.22222	100	98.95833	100	97.22222	100	98.95833		97.22222	100	98.95833	23.45679
**P34**	36	F	28	9	10	10		3.5	58.97436	100	100	83.33333	76.92308	100	100	90.625					19.26531
**P35**	67	F	48	9	16	16		3.5													
**P37**	24	M	23	8	16	16		2.5	89.74359	100	90.47619	93.75	89.74359	100	90.47619	93.75					29.66797
**P38**	73	M	40	10	17	17		3	94.87179	100	100	97.91667	94.87179	100	100	97.91667					
**P39**	45	F	14	7	6	6		3.5	35.89744	80.55556	95.2381	65.625	30.76923	80.55556	90.47619	62.5					19.53125
**P40**	39	M	14	6	20	20		1.5	100	100	100	100	100	100	100	100					22.72044
**P41**	45	M	27	7	16	16		3													
**P49**	24	M	16	6	20	20		1.5	94.87179	100	100	97.91667	94.87179	100	100	97.91667	94.87179	100	100	97.91667	24

**P50**	59	F	55	6	12	10	10	4	64.10256	97.22222	100	84.375	38.46154	91.66667	95.2381	70.83333	38.46154	91.66667	95.2381	70.83333	21.45329

Abbreviations: RUs: number of repeat units; MRCtotal T1: MRC sumscore at Time 1 (baseline); MRCtotal T2: MRC sumscore at Time 2 (median time between T2 and T1 was 12.5 months (12 – 15.5)); MRCtotal T3: MRC sumscore at Time 3 (median time between T3 and T2 was 13.5 months (12 – 20.5); CSS: Clinical Severity Score; MFM: Motor Function Measurement; MFMD1 : dimension D1 of MFM; MFM D2 : dimension D2 of MFM; MFM D3 : dimension D3 of MFM; MFMtotal= total MFM sum score; MFMD2T1: MFMD2, Time T1; MFMD3T1: MFMD3, Time T1; MFMtotalT1: MFMtotal, Time T1; MFMD1T2: MFMD1, Time T2; MFMD2T2: MFMD2, Time T2; MFMD3T2: MFMD3, Time T2; MFMtotalT2: MFMtotal, Time T2; MFMD1T3: MFMD1, Time T3; MFMD2T3: MFMD2, Time T3; MFMD3T3: MFMD3, Time T3; MFMtotalT3: MFMtotal, Time T3; BMI: Body Mass Index
