# Supplementary material for: Long-term follow-up of MRI changes in thigh muscles of patients with Facioscapulohumeral dystrophy: A quantitative study
Source: PLoS One. 2017 Aug 25;12(8):e0183825. doi: 10.1371/journal.pone.0183825 (PMC5571945; doi:10.1371/journal.pone.0183825)
Supplement: S2 Table — (PDF) [file pone.0183825.s002.pdf]

	MPI_{tot} T1	MPI_{tot} T2	MPI_{tot} T3
**P1**	75.60467	78.01136	80.07146
**P3**	33.70199	35.63093	33.96884
**P4**	32.22896	32.03829	
**P7**	48.68576	50.4535	50.96224
**P8**	33.60427	34.98727	37.92692
**P10**	37.5931	35.83063	
**P11**	37.26376	36.91809	
**P12**	59.81444	63.00822	
**P14**	40.61247	40.8312	
**P15**	60.2025	61.09186	60.20196
**P16**	39.58293	43.3677	
**P17**	30.76724	30.90592	
**P18**	54.60558	55.82067	
**P19**	58.58734	60.44241	61.38027
**P20**	79.77439	79.39049	80.25425
**P21**	78.95318	78.661	
**p23**	32.38672	32.94999	35.30371
**P25**	48.38841	51.53739	
**P26**	43.82345	43.66411	
**P27**	68.03108	70.90136	
**P28**	54.11972	56.06815	
**P29**	35.10943	41.08331	
**P30**	55.43668	58.24664	
**P31**	43.16568	45.66433	
**P32**	35.66399	35.68426	35.50563
**P33**	31.74293	31.75828	31.89914
**P34**	37.73865	39.94288	
**P35**	50.16305	51.93222	
**P37**	36.12435	37.87405	
**P38**	38.1822	39.94288	
**P39**	73.14263	74.07786	
**P40**	36.00926	37.85528	
**P41**	32.22758	32.07574	
**P49**	29.7118	30.49351	
**P50**	37.43986	37.65205	39.12665

Abbreviations: MPI_{total}: mean pixel intensity. T1 refers to baseline, T2 to 12.5 months (12 – 15.5) after T1 and T3 to 13.5 months (12 – 20.5) after T2.
